# Efficacy and Tolerability of Intravenous Ferric Carboxymaltose in Patients with Iron Deficiency at a Hospital Outpatient Clinic: A Retrospective Cohort Study of Real-World Clinical Practice

**DOI:** 10.1155/2017/3106890

**Published:** 2017-07-03

**Authors:** António Robalo Nunes, Ana Palricas Costa, Sara Lemos Rocha, Ana Garcia de Oliveira

**Affiliations:** ^1^Hospital de Dia de Imuno-Hemoterapia, Hospital Pulido Valente, Centro Hospitalar Lisboa Norte (CHLN), Alameda das Linhas de Torres 117, 1769-001 Lisboa, Portugal; ^2^Hospital de Dia de Imuno-Hemoterapia, Hospital de Santa Maria, CHLN, Avenida Prof. Egas Moniz, 1649-035 Lisboa, Portugal

## Abstract

Ferric carboxymaltose (FCM) is an intravenous iron formulation to correct iron deficiency. Although its use has been extensively studied in clinical trials, real-world evidence regarding FCM treatment is scarce. Our aim was to evaluate the efficacy and tolerability of FCM treatment in patients with iron deficiency, with or without anemia, at a hospital outpatient clinic. Data was collected retrospectively from medical records. During this 2-year study, 459 patients were included. Mean age was 58.6 ± 17.5 years and most patients received cumulative FCM doses of 501–1000 mg (63.2%). Six weeks after administration of FCM, efficacy endpoints hemoglobin increase ≥2 g/dL, hemoglobin increase ≥3 g/dL, and transferrin saturation > 20% were attained by 41%, 20%, and 63% of patients, respectively. Patients who received higher FCM doses showed significant reduced odds of not achieving hemoglobin increase ≥2 g/dL (501–1000 mg, adjusted odds ratio [OR]: 0.34, 95% confidence interval [CI] 0.18–0.62; 1001–3000 mg, OR: 0.19, 95% CI 0.07–0.49), compared to 500 mg doses. Treatment-emergent adverse events were documented in <4% of patients. In conclusion, FCM treatment was effective and well-tolerated by outpatients with iron deficiency at a hospital clinic, and its dosage should be adjusted to improve iron deficiency management in clinical practice.

## 1. Introduction

Iron deficiency is the most common nutritional deficiency worldwide. It has been estimated to affect at least 30–40% of preschool children and pregnant women and about one-sixth of the total population in developed countries [1–3]. Iron deficiency is frequently associated with fatigue, susceptibility to infections, and poorer work capacity and quality of life and is a major cause for anemia in both developing and developed countries [1, 2]. Moreover, comorbidity of iron deficiency and anemia with other diseases have been described to increase the risk of morbidity and mortality [2, 4].

Iron deficiency can occur in two main types: absolute, associated with low total body iron stores due to decreased dietary intake or blood loss, or functional, which occurs when the iron absorption or release from storage areas is inadequate, despite normal or increased total body iron stores [4–6]. Functional iron deficiency may be caused by chronic inflammation, associated with several conditions such as renal failure, congestive heart failure, inflammatory bowel disease, cancer, or autoimmune conditions, which contributes to the onset of the anemia of chronic diseases [7, 8].

Treatment for iron deficiency involves the restoration of iron stores to normal levels and the increase of hemoglobin values, as well as addressing its underlying causes [9]. Oral iron supplementation is the first-line therapy for iron deficiency [6]. However, oral iron therapy presents disadvantages, such as low absorption of iron, drug interactions, increased oxidative stress in the gastrointestinal tract, or high incidence of gastrointestinal adverse events, which can reduce patient compliance [4]. Thus, oral iron has limited efficacy in treating chronic iron deficiency anemia (IDA), hence being more adequate for short periods of iron requirements [6, 10]. Intravenous iron formulations such as ferric carboxymaltose, iron dextran, ferric gluconate, and iron sucrose may potentially solve these issues and may be more convenient both to healthcare providers and patients, if oral iron therapy does not provide the required correction of iron deficiency or is not well-tolerated by patients [4, 6, 9].

Ferric carboxymaltose (FCM) is a relatively recent intravenous iron formulation, which can be administered in short periods of time and at large single doses (up to 1000 mg) [4, 11]. Its efficacy in correcting iron deficiency has been evaluated in several adult populations, namely, patients with gastrointestinal disorders, chronic kidney disease, chronic heart failure, gynecological and obstetrics disorders, and neoplasms [4, 5, 7–10, 12]. Indeed, these studies highlighted the advantages of using intravenous FCM in detriment of oral iron, in terms of both efficacy and tolerability [4, 8–10, 12].

In clinical trials, FCM was associated with rapid hematopoietic improvement by increasing hemoglobin levels, refurnishing iron stores (i.e., changes in ferritin levels), and increasing available iron for erythropoiesis (i.e., raise in transferrin saturation) [9, 12]. Administration of FCM was also well-tolerated by patients, with most drug-related adverse events considered to be mild to moderate in severity [4, 10, 12]. However, real-world data on the efficacy of intravenous FCM therapeutics in current clinical practice are still scarce [5, 8, 13, 14]. Therefore, the purpose of this study was to evaluate the efficacy and tolerability of intravenous FCM treatment of iron deficiency, in patients with or without anemia, attending an outpatient hospital clinic setting.

## 2. Materials and Methods

### 2.1. Study Design

This was a retrospective cohort study, conducted at the Immunohemotherapy (transfusion medicine) outpatient clinic of Hospital Pulido Valente and Hospital de Santa Maria, both hospitals of Centro Hospitalar Lisboa Norte. Data was collected retrospectively from medical records on all consecutive patients with iron deficiency attending the hospital outpatient clinic, who were treated with intravenous FCM from January 2014 to December 2015. For each patient included in this study, data was only collected regarding treatment of first episode with a follow-up period of six weeks (±1 week), and no subsequent episodes were included.

### 2.2. Patients

Eligible patients were adult individuals (≥18 years old) who had iron deficiency with or without anemia and were submitted to intravenous FCM therapeutics at the hospital outpatient clinic. Iron deficiency was defined as transferrin saturation <20% [15]. Anemia was defined as hemoglobin ≤12 g/dL in women, and ≤13 g/dL in men, according to the World Health Organization threshold hemoglobin concentrations [1]. Patients were excluded if they had missing data regarding any of the hematological or iron laboratory tests or did not complete the first episode treatment with FCM.

Patients were assigned to FCM dose levels according to their individual potential iron requirement, as recommended by the Summary of Product Characteristics (SmPC) or calculated using the Ganzoni formula [16]. According to this formula, individual potential iron requirement is a function of the patient's hemoglobin level and body weight: total iron deficit (mg) = body weight (kg) × (target hemoglobin − actual hemoglobin) (g/dL) × 2.4 + depot iron of 500 mg.

Patients received single doses of FCM from 500 mg or 15 mg/kg up to a maximum dose of 1000 mg per week, given as intravenous infusions over 15 to 30 min.

### 2.3. Data Collection

Demographic, clinical, laboratory, and safety data were retrospectively collected from patients' medical records, at admission (before treatment) and follow-up visits (after treatment). Demographic data included age and gender, while clinical data included primary diagnosis, cumulative iron dose levels, and adverse events. Primary diagnoses were coded using the high-level classification of the 10th revision of the International Statistical Classification of Diseases and Related Health Problems (ICD-10) [17]. Patients were stratified by cumulative iron dose levels in three categories: 500 mg, 501–1000 mg, and 1001–3000 mg. Laboratory test results data included pretreatment and posttreatment serum hemoglobin, hematocrit, mean corpuscular volume (MCV), mean corpuscular hemoglobin (MCH), ferritin, and transferrin saturation. Reference ranges of the local laboratory were 12.0–15.3 g/dL (female) or 13.0–17.5 g/dL (male) for hemoglobin; 36–46% (female) or 40–50% (male) for hematocrit; 80–97 fL (female or male) for MCV; 27–33 pg (female or male) for MCH; and 13–150 ng/mL (female) or 30–400 ng/mL (male) for ferritin.

### 2.4. Endpoints

The primary efficacy endpoint of the data analysis was the proportion of patients with a hemoglobin increase of ≥2.0 g/dL after treatment (i.e., achieving a hematopoietic response). Secondary efficacy endpoints were the proportion of patients attaining a hemoglobin increase of ≥3.0 g/dL after treatment, and the proportion of patients attaining a transferrin saturation >20% after treatment (i.e., achieving an iron bioavailability response).

Safety endpoint was the incidence of treatment-emergent adverse events (TEAEs) occurring during or after administration of intravenous FCM. All safety data were collected during clinical care, without a systematic method, which means some data might not have been registered in the patient files.

### 2.5. Statistical Analysis

Continuous variables were summarized by mean and standard deviation or median and 1st quartile-3rd quartile as applicable. Categorical variables were summarized by relative and absolute frequencies. The variation in the hematological and iron parameters was assessed using the paired* t*-test or Wilcoxon signed-rank test as applicable.

Primary diagnoses were coded using the high-level classification of ICD-10. For the analyses of the efficacy endpoints, classes with less than 10 patients were merged into a single class named “other diseases,” to ensure an adequate statistical power for the comparisons. The remaining classes (i.e., neoplasms, diseases of the genitourinary system, diseases of the circulatory system, and diseases of the digestive system) were analyzed individually.

Due to the study design, no reference group was established. Therefore, pairwise exploratory comparisons regarding diagnoses groups were performed against all the remaining subjects (e.g., the group of patients with neoplasms was compared against the group of subjects who did not have neoplasms). As for the dosage groups, pairwise exploratory comparisons were performed against the lower dose (i.e., 500 mg), used as reference dose.

To assess the treatment efficacy, a logistic regression model was used to determine the probability of clinical failure in each endpoint (i.e., not achieving the efficacy endpoint after treatment with intravenous FCM). The odds ratio (OR) and 95% confidence intervals (CIs) were estimated. The computed OR were adjusted for age and pretreatment hemoglobin for the endpoints related to hemoglobin increase and for age and pretreatment transferrin saturation for the endpoint related to the increase of transferrin saturation.

Due to the low incidence of adverse events, no statistical inference was performed.

## 3. Results

### 3.1. Patients Characteristics

A total of 459 patients who received intravenous FCM treatment were included in the study. Demographic and clinical characteristics of the patients are shown in Table 1. Mean age was 58.6 ± 17.5 years and most patients were female (78%). In all, anemia was recorded in 82.4% of patients, iron deficiency was recorded in 98% of patients, and IDA was recorded in 81.3% of patients. The majority of patients received a single FCM dose between 501 mg and 1000 mg (63.2%). Only 10.2% of patients received cumulative iron doses from 1001 mg to 3000 mg in divided doses (one per week). Most common primary diagnoses were diseases of the digestive system (43.4%), diseases of the genitourinary system (26.4%), neoplasms (10.2%), and diseases of the circulatory system (8.3%). Other diseases comprised the following categories: diseases of the blood and blood-forming organs and certain disorders involving the immune mechanism (3.3%); endocrine, nutritional, and metabolic diseases (2.0%); diseases of the musculoskeletal system and connective tissue (1.7%); diseases of the nervous system (1.5%); diseases of the eye and adnexa (1.3%); pregnancy, childbirth, and the puerperium (1.1%); diseases of the respiratory system (0.7%); and certain infectious and parasitic diseases (0.2%). After 6 weeks (±1 week) following FCM treatment, all hematological and iron measurements significantly increased from baseline (Table 2).

### 3.2. Efficacy Endpoints

The primary (i.e., hemoglobin increase ≥2 g/dL) and secondary (i.e., hemoglobin increase ≥3 g/dL and transferrin saturation > 20%) efficacy endpoints, following intravenous FCM treatment, are shown in Table 3. After 6 weeks, hemoglobin increase of ≥2 g/dL was attained by 41% of all patients, 40% in the IDA group, 40% in the diseases of the digestive system group, 55% in the diseases of the genitourinary system group, 26% in the neoplasms group, and 29% in the diseases of the circulatory system group. Moreover, our analysis indicated that patients with diseases of the genitourinary system presented significant decreased odds of clinical failure (OR: 0.46, 95% CI 0.25, 0.87), whereas patients with diseases of the circulatory system showed about three times higher odds of clinical failure (OR: 3.34, 95% CI 1.31–8.98). Regarding cumulative FCM treatment dose, we found significant decreased odds of clinical failure in patients who received doses ranging from 501 to 1000 mg (OR: 0.34, 95% CI 0.18–0.62) or 1001–3000 mg doses (OR: 0.19, 95% CI 0.07–0.49), compared to patients who received doses of 500 mg (Table 3).

Hemoglobin increase of ≥3 g/dL after 6 weeks after FCM dose was attained by 20% of all patients, 24% of the patients in the IDA group, 22% in the diseases of the digestive system group, 26% in the diseases of the genitourinary system group, 11% in the neoplasms group, and 16% in the diseases of the circulatory system group. Furthermore, our analysis indicated that patients with IDA presented significant lower odds of clinical failure (OR: 0.07, 95% CI 0.01–0.24). Concerning total FCM treatment dose, our analysis showed significant lower odds of clinical failure in patients who received doses ranging from 501 to 1000 mg (OR: 0.36, 95% CI 0.12–0.92) or 1001–3000 mg doses (OR: 0.23, 95% CI 0.06–0.73), compared to patients who received doses of 500 mg (Table 3).

Finally, transferrin saturation > 20% after 6 weeks after FCM dose was attained by 63% of all patients, 69% of patients in both IDA and iron deficiency without anemia groups, 63% in the diseases of the digestive system group, 68% in the diseases of the genitourinary system group, 67% in the neoplasms group, and 47% in the diseases of the circulatory system group. Our analysis indicated significant lower odds of clinical failure in patients who received doses ranging from 501 to 1000 mg (OR: 0.57, 95% CI 0.36–0.88) or 1001–3000 mg doses (OR: 0.25, 95% CI 0.10–0.55), compared to patients who received doses of 500 mg (Table 3).

### 3.3. Safety Endpoint

A total of 17 TEAEs were recorded in 15 (3.3%) patients. The most common documented TEAEs were nausea (*n* = 4), rash (*n* = 4), and urticaria (*n* = 2). Additionally, patients also experienced diarrhea (*n* = 1), chill (*n* = 1), myalgia (*n* = 1), abdominal pain (*n* = 1), wheeze (*n* = 1), arthralgia (*n* = 1), and hyperthermia (*n* = 1).

## 4. Discussion

This study evaluated the efficacy and tolerability of intravenous FCM treatment for the correction of iron deficiency, with or without anemia, in patients with diverse diagnoses, who were attending a hospital outpatient Immunohemotherapy clinic. The rationale for studying the complete population of patients under FCM treatment and not only some subgroups as most studies report [8, 14, 18–21] was to improve the prescription standards of the intravenous FCM in clinical practice.

Treatment efficacy with FCM considering the primary endpoint of hemoglobin increase ≥2 g/dL at week 6 was 41% in all patients, in agreement with previous studies performed in various populations of patients with IDA [4, 5, 8, 9]. For patients with IDA, no differences in treatment efficacy were found for hemoglobin increase of ≥2 g/dL and transferrin saturation > 20%, compared with patients without IDA. However, we found a significant difference in hemoglobin increase of ≥3 g/dL. This finding was expected, as achieving such a high increase is more challenging for patients with normal hemoglobin levels.

Efficacy of FCM was lower in patients with neoplasms and diseases of the circulatory system. This might be due to the increased inflammatory states commonly caused by these diseases (e.g., cancer, acute or chronic congestive heart failure), which are known to impair patients' ability to absorb iron and hence to achieve a rapid hemoglobin increase [8]. To illustrate, neoplasms patients achieved lower efficacy in hemoglobin increase, but higher efficacy in transferrin saturation >20%. This finding is consistent with the presence of anemia of chronic disease rather than absolute iron deficiency (i.e., low body iron stores), which makes these patients less respondent to iron supplementation [7, 8, 11, 22]. In contrast, FCM efficacy was significantly higher in patients with diseases of the genitourinary system for hemoglobin increase, as most of the diagnoses included in this group were heavy uterine bleeding, a condition that presents a good response to iron treatment due to its pathophysiology [5, 8, 10, 21, 22]. Accordingly, male patients presented an apparent lower efficacy rate, although not significant. Finally, efficacy of FCM treatment in patients with diseases of the digestive system was comparable to the ones of other subgroups. This finding is consistent with the pathophysiology of most diseases in this subgroup, which include either food disorders or acute bleedings. Other diseases had a low presence to allow any conclusion.

The incidence of TEAEs was low in this study (<4%). Rash and nausea were the most common TEAEs, and no serious adverse events were found in the medical records. Although these events might be underreported, it was not expected that FCM would present a high toxicity, as other studies also reported FCM as a well-tolerated therapy [6, 9–12, 23, 24] with an acceptable safety profile in patients with diverse diagnoses [5, 8, 10, 11, 23]. Nevertheless, the conservativeness in the cumulative FCM treatment doses might also be a factor that explains the low incidence of TEAEs.

Not so surprisingly, higher cumulative doses of FCM (i.e., 501–1000 mg and 1001–3000 mg) showed a significant higher efficacy compared to the lower dose (i.e., 500 mg), used as reference. In this study, doses of 500 mg were on average 75% less effective than doses above 1000 mg, even when adjusted for age and predose hemoglobin or transferrin saturation serum levels. Although no data on body weight was collected, we may assume that patients were frequently treated with lower doses than the ones recommended in the SmPC due to the high frequency of cumulative FCM doses of 500 mg (26.6%). This lower dosage profile might affect overall treatment efficacy in our clinical setting, as demonstrated by the lower efficacy rates associated with lower cumulative FCM doses shown in this study.

Although most clinical trials and studies reported low toxicity for FCM, clinicians might be still using the traditionally lower doses of oral iron therapy, which is less tolerated by the patients [6, 9–12, 23, 24]. Therefore, our results suggest that patients might be undertreated due to treatment conservativeness of the clinicians prescribing intravenous iron therapeutics. Indeed, we found better hematological responses with the use of higher cumulative FCM doses. Hence, our study provides additional data supporting the indication of higher cumulative FCM doses for the treatment of iron deficiency, across patients with diverse diagnoses. As previously reported by other authors [10, 18, 22, 23, 25], higher FCM doses may benefit the patients in terms of treatment efficacy and quality of life, without significantly impacting treatment tolerability and safety. In addition, administration of single large doses up to 1000 mg, instead of multiple lower iron dosages, is more convenient for the patients and may result in cost savings for healthcare systems and society [5, 8].

To our knowledge, this is one of the first observational studies reporting the efficacy of intravenous FCM treatment in a real-world setting, across a population of patients with a considerable dimension (*n* = 459) [5, 8, 13, 14]. Moreover, this study presents data on a single drug formulation, prescribed to patients with diverse ages and primary diagnoses. Therefore, we may assume that the results of this study are representative of FCM treatment efficacy in patients with iron deficiency in current clinical practice. Nevertheless, due to its retrospective and observational nature, this study presents some limitations. First, our analyses were based on the clinical and hematological data collected during routine clinical practice, which might have led to an apparent underreporting of adverse events and exclusion of some valid cases. Moreover, as several clinicians were involved in the prescription of FCM dosage, some patients were treated as recommended by the SmPC, while other patients were treated according to the Ganzoni formula. In addition, the lack of a clearly defined reference group limited our conclusions regarding the overall FCM treatment efficacy. Therefore, findings of this study, although valid, may only be considered as exploratory. Additional prospective studies should be performed, using appropriate populations and systematic documentation of hematological and TEAEs data. Future research should also address the efficacy and safety of intravenous FCM in different real-world clinical settings.

## 5. Conclusions

Our study showed that the use of FCM increases hemoglobin levels ≥2 g/dL after 6 weeks in 41% of all iron deficiency patients, with or without anemia, in a hospital day setting and was associated with a low incidence of TEAEs. Moreover, treatment efficacy was significantly higher in patients who received higher cumulative FCM doses, that is, 501–1000 and 1001–3000 mg, compared to patients who received doses of 500 mg. Our findings indicate that intravenous FCM dosage should be adjusted to improve treatment efficacy of iron deficiency in different clinical practice settings.

## Figures and Tables

**Table 1:** Demographic and clinical characteristics of the study population.

Characteristic	Patients (*N *= 459)
Age (years), mean (SD)	58.6 (17.5)
Elderly > 65 years, *n* (%)	187 (40.7)
Male, *n* (%)	101 (22.0)
Anemia, *n* (%)	378 (82.4)
Iron deficiency, *n* (%)	450 (98.0)
Iron deficiency anemia, *n* (%)	373 (81.3)
Iron deficiency without anemia, *n* (%)	77 (16.8)
Cumulative FCM treatment dose, *n* (%)	
500 mg	122 (26.6)
501–1000 mg	290 (63.2)
1001–3000 mg	47 (10.2)
Diagnosis per ICD-10 high level category, *n* (%)	
Diseases of the digestive system	199 (43.4)
Diseases of the genitourinary system	121 (26.4)
Neoplasms	47 (10.2)
Diseases of the circulatory system	38 (8.3)
Other diseases	54 (11.8)

FCM: ferric carboxymaltose; ICD-10: International Classification of Diseases 10th Revision.

**Table 2:** Hematological laboratory tests before and after treatment with intravenous ferric carboxymaltose (FCM).

Variables	Before treatment	After treatment	*p* value
Hemoglobin (g/dL)	10.67 (1.67)	12.65 (1.26)	<0.001
Hematocrit (%)	33.32 (4.48)	38.57 (3.81)	<0.001
MCV (fL)	80.67 (8.94)	86.75 (6.56)	<0.001
MCH (pg)	25.62 (3.63)	28.30 (2.37)	<0.001
Ferritin (*μ*g/dL)	9.60 (5.70–24.00)	137.00 (59.95–243.10)	<0.001
Transferrin saturation (%)	8.70 (4.76)	25.02 (10.16)	<0.001

Values are expressed as mean (standard deviation) or median (1st quartile-3rd quartile). Laboratory reference ranges in normal adults: hemoglobin, 12.0–15.3 g/dL (female) and 13.0–17.5 g/dL (male); hematocrit, 36–46% (female) and 40–50% (male); MCV, 80–97 fL; MCH, 27–33 pg; ferritin, 13–150 *μ*g/dL (female) and 30–400 *μ*g/dL (male); MCH: mean corpuscular hemoglobin; MCV: mean corpuscular volume.

**Table 3:** Odds ratio of clinical failure in primary and secondary efficacy endpoints for intravenous FCM treatment.

Characteristic	Hemoglobin increase ≥2 g/dL		Hemoglobin increase ≥3 g/dL		Transferrin saturation >20%^a^	
	*n* (%)	OR (95% CI)	*n* (%)	OR (95% CI)	*n* (%)	OR (95% CI)
All	190 (41)	—	94 (20)	—	285 (63)	—
Male	35 (35)	1.7 (0.94–3.13)	20 (20)	1.01 (0.50–2.10)	54 (55)	1.45 (0.91–2.31)
Iron deficiency anemia	184 (40)	1.27 (0.42–3.45)	92 (24)	**0.07 (0.01**–**0.24)**	53 (69)	1.29 (0.77–2.23)
Iron deficiency without anemia	—	—	—	—	53 (69)	0.77 (0.45–1.30)
Diseases of the digestive system	79 (40)	0.88 (0.54–1.42)	43 (22)	0.57 (0.31–1.04)	123 (63)	0.95 (0.63–1.4)
Diseases of the genitourinary system	67 (55)	**0.46 (0.25**–**0.87)**	31 (26)	1.22 (0.59–2.54)	82 (68)	0.88 (0.52–1.48)
Neoplasms	12 (26)	2.19 (0.98–5.19)	5 (11)	1.63 (0.57–5.72)	30 (67)	0.70 (0.35–1.38)
Diseases of the circulatory system	11 (29)	**3.34 (1.31**–**8.98)**	6 (16)	2.1 (0.72–6.86)	18 (47)	1.82 (0.91–3.65)
Other diseases	21 (40)	0.94 (0.45–1.97)	9 (17)	1.12 (0.47–2.90)	32 (62)	1.13 (0.61–2.06)
Cumulative FCM treatment dose^b^						
500 mg	18 (15)	—	5 (4)	—	58 (51)	—
501–1000 mg	141 (49)	**0.34 (0.18**–**0.62)**	70 (24)	**0.36 (0.12**–**0.92)**	189 (65)	**0.57 (0.36**–**0.88)**
1001–3000 mg	31 (66)	**0.19 (0.07**–**0.49)**	19 (40)	**0.23 (0.06**–**0.73)**	38 (81)	**0.25 (0.10**–**0.55)**

Significant odds ratio in boldface; CI: confidence interval; FCM: ferric carboxymaltose; OR: odds ratio; ^a^*N* = 450, only patients who had transferrin saturation <20% before treatment were considered for this endpoint. ^b^Reference group: cumulative iron dose of 500 mg.
