# Corrigendum to “Efficacy and Tolerability of Intravenous Ferric Carboxymaltose in Patients with Iron Deficiency at a Hospital Outpatient Clinic: A Retrospective Cohort Study of Real-World Clinical Practice”

**DOI:** 10.1155/2019/9242607

**Published:** 2019-01-01

**Authors:** António Robalo Nunes, Ana Palricas Costa, Sara Lemos Rocha, Ana Garcia de Oliveira

**Affiliations:** ^1^Hospital de Dia de Imuno-Hemoterapia, Hospital Pulido Valente, Centro Hospitalar Lisboa Norte (CHLN), Alameda das Linhas de Torres 117, 1769-001 Lisboa, Portugal; ^2^Hospital de Dia de Imuno-Hemoterapia, Hospital de Santa Maria, CHLN, Avenida Prof. Egas Moniz, 1649-035 Lisboa, Portugal

In the article titled “Efficacy and Tolerability of Intravenous Ferric Carboxymaltose in Patients with Iron Deficiency at a Hospital Outpatient Clinic: A Retrospective Cohort Study of Real-World Clinical Practice” [1], there were the following errors.

(i) In the Statistical Analysis section, “The computed OR were adjusted for age and pretreatment hemoglobin for the endpoints related to hemoglobin increase and for age and pretreatment transferrin saturation for the endpoint related to the increase of transferrin saturation” should be corrected to “The computed OR were adjusted for age and pre-treatment hemoglobin for the endpoints related with hemoglobin increase, and for age and pre-treatment ferritin for the endpoint related with the increase of transferrin saturation.”

(ii) There were multiple errors in the Efficacy Endpoints section. The corrected section is as follows.

“The primary (i.e., hemoglobin increase ≥2 g/dL) and secondary (i.e., hemoglobin increase ≥3 g/dL and transferrin saturation >20%) efficacy endpoints, following intravenous FCM treatment, are shown in Table 3. After 6 weeks, hemoglobin increase ≥2 g/dL was attained by 41% of all patients, 49% in the IDA group, 40% in the diseases of the digestive system group, 55% in the diseases of the genitourinary system group, 26% in the neoplasms group, and 29% in the diseases of the circulatory system group. Moreover, our analysis indicated that patients with IDA or diseases of the genitourinary system presented significant decreased odds of clinical failure (OR: 0.07, 95% CI 0.03-0.15 or OR: 0.46, 95% CI 0.25-0.87, respectively), whereas patients with diseases of the circulatory system showed about three times higher odds of clinical failure (OR: 3.34, 95% CI 1.31-8.98). Regarding cumulative FCM treatment dose, we found significant decreased odds of clinical failure in patients who received doses ranging from 501 to 1000 mg (OR: 0.34, 95% CI 0.18-0.62) or 1001-3000 mg doses (OR: 0.19, 95% CI 0.07-0.49), compared to patients who received doses of 500 mg (Table 3).

Hemoglobin increase of ≥3 g/dL after 6 weeks after FCM dose was attained by 20% of all patients, 25% of the patients in the IDA group, 22% in the diseases of the digestive system group, 26% in the diseases of the genitourinary system group, 11% in the neoplasms group, and 16% in the diseases of the circulatory system group. Furthermore, our analysis indicated that patients with IDA presented significant lower odds of clinical failure (OR: 0.07, 95% CI 0.01-0.22). Concerning total FCM treatment dose, our analysis showed significant lower odds of clinical failure in patients who received doses ranging from 501 to 1000 mg (OR: 0.36, 95% CI 0.12-0.92) or 1001-3000 mg doses (OR: 0.23, 95% CI 0.06-0.73), compared to patients who received doses of 500 mg (Table 3).

Finally, transferrin saturation >20% after 6 weeks after FCM dose was attained by 63% of all patients, 62% of patients in the IDA group, 69% of patients in the iron deficiency without anemia group, 63% in the diseases of the digestive system group, 68% in the diseases of the genitourinary system group, 67% in the neoplasms group, and 47% in the diseases of the circulatory system group. Our analysis indicated significant lower odds of clinical failure in patients who received doses ranging from 501 to 1000 mg (OR: 0.57, 95% CI 0.36-0.88) or 1001-3000 mg doses (OR: 0.25, 95% CI 0.10-0.55), compared to patients who received doses of 500 mg (Table 3).”

(iii) There were errors in Table 3. The corrected table is shown below.

(iv) In the Discussion section, “For patients with IDA, no differences in treatment efficacy were found for hemoglobin increase of ≥2 g/dL and transferrin saturation > 20%, compared with patients without IDA. However, we found a significant difference in hemoglobin increase of ≥3 g/dL” should be corrected to “For patients with IDA, no differences in treatment efficacy were found for transferrin saturation >20%, compared with patients without IDA. However, we found significant differences in both hemoglobin-related efficacy endpoints.”

## Supplementary Materials

Supplementary Materials The underlying patient-level data and a script with all the logistic regression models adjusted to generate the Odds Ratio and respective confidence intervals for Table 3 are included as Supplementary Materials.

## Figures and Tables

**Table 3:** Odds ratio of clinical failure in primary and secondary efficacy endpoints for intravenous FCM treatment.

	Hemoglobin increase ≥2 g/dL			Hemoglobin increase ≥3 g/dL			Transferrin saturation >20%^a^		
Characteristic	N	n (%)	OR (95% CI)	N	n (%)	OR (95% CI)	N	n (%)	OR (95% CI)
All	459	190 (41)	-	459	94 (20)	-	450	285 (63)	-
Male	101	35 (35)	1.7 (0.94-3.13)	101	20 (20)	1.01 (0.50-2.10)	98	54 (55)	1.45 (0.91-2.31)
Iron deficiency anemia	373	184 (49)	**0.07 (0.03-0.15)**	373	92 (25)	**0.07 (0.01-0.22)**	373	232 (62)	1.29 (0.77-2.23)
Iron deficiency without anemia	77	-	-	77	-	-	77	53 (69)	0.77 (0.45-1.30)
Diseases of the digestive system	199	79 (40)	0.88 (0.54-1.42)	199	43 (22)	0.57 (0.31-1.04)	195	123 (63)	0.95 (0.63-1.4)
Diseases of the genitourinary system	121	67 (55)	**0.46 (0.25-0.87)**	121	31 (26)	1.22 (0.59-2.54)	52	82 (68)	0.88 (0.52-1.48)
Neoplasms	47	12 (26)	2.19 (0.98-5.19)	47	5 (11)	1.63 (0.57-5.72)	45	30 (67)	0.70 (0.35-1.38)
Diseases of the circulatory system	38	11 (29)	**3.34 (1.31-8.98)**	38	6 (16)	2.1 (0.72-6.86)	38	18 (47)	1.82 (0.91-3.65)
Other diseases	54	21 (39)	0.94 (0.45-1.97)	54	9 (17)	1.12 (0.47-2.90)	52	32 (62)	1.13 (0.61-2.06)
Cumulative FCM treatment dose^b^									
500 mg	122	18 (15)	-	122	5 (4)	-	114	58 (51)	-
501–1000 mg	290	141 (49)	**0.34 (0.18-0.62)**	290	70 (24)	**0.36 (0.12-0.92)**	289	189 (65)	**0.57 (0.36-0.88)**
1001–3000 mg	47	31 (66)	**0.19 (0.07-0.49)**	47	19 (40)	**0.23 (0.06-0.73)**	47	38 (81)	**0.25 (0.10-0.55)**

Significant odds ratio in boldface.

CI: confidence interval; FCM: ferric carboxymaltose; N: total number of subjects; n: number of subjects achieving the endpoint; OR: odds ratio.

^a^ Only patients who had transferrin saturation <20% before treatment were considered for this endpoint. Therefore, all data presented for this endpoint only considers those subjects with iron deficiency.

^b^ Reference group: cumulative iron dose of 500 mg.
